# A Fusion Biopsy Framework for Prostate Cancer Based on Deformable Superellipses and nnU-Net

**DOI:** 10.3390/bioengineering9080343

**Published:** 2022-07-26

**Authors:** Nicola Altini, Antonio Brunetti, Valeria Pia Napoletano, Francesca Girardi, Emanuela Allegretti, Sardar Mehboob Hussain, Gioacchino Brunetti, Vito Triggiani, Vitoantonio Bevilacqua, Domenico Buongiorno

**Affiliations:** 1 Department of Electrical and Information Engineering (DEI), Polytechnic University of Bari, 70126 Bari, BA, Italy; antonio.brunetti@poliba.it (A.B.); v.napoletano3@studenti.poliba.it (V.P.N.); f.girardi@studenti.poliba.it (F.G.); e.allegretti@studenti.poliba.it (E.A.); sardarmehboob.hussain@poliba.it (S.M.H.); vitoantonio.bevilacqua@poliba.it (V.B.); domenico.buongiorno@poliba.it (D.B.); 2 Apulian Bioengineering s.r.l., Via delle Violette n.14, 70026 Modugno, BA, Italy; 3 Masmec Biomed SpA, Via delle Violette n.14, 70026 Modugno, BA, Italy; gioacchino.brunetti@masmecbiomed.com (G.B.); vito.triggiani@masmecbiomed.com (V.T.)

**Keywords:** deformable superellipses, TRUS, prostate segmentation, MRI-targeted biopsy

## Abstract

In prostate cancer, fusion biopsy, which couples magnetic resonance imaging (MRI) with transrectal ultrasound (TRUS), poses the basis for targeted biopsy by allowing the comparison of information coming from both imaging modalities at the same time. Compared with the standard clinical procedure, it provides a less invasive option for the patients and increases the likelihood of sampling cancerous tissue regions for the subsequent pathology analyses. As a prerequisite to image fusion, segmentation must be achieved from both MRI and TRUS domains. The automatic contour delineation of the prostate gland from TRUS images is a challenging task due to several factors including unclear boundaries, speckle noise, and the variety of prostate anatomical shapes. Automatic methodologies, such as those based on deep learning, require a huge quantity of training data to achieve satisfactory results. In this paper, the authors propose a novel optimization formulation to find the best superellipse, a deformable model that can accurately represent the prostate shape. The advantage of the proposed approach is that it does not require extensive annotations, and can be used independently of the specific transducer employed during prostate biopsies. Moreover, in order to show the clinical applicability of the method, this study also presents a module for the automatic segmentation of the prostate gland from MRI, exploiting the nnU-Net framework. Lastly, segmented contours from both imaging domains are fused with a customized registration algorithm in order to create a tool that can help the physician to perform a targeted prostate biopsy by interacting with the graphical user interface.

## 1. Introduction

**Figure 1:**
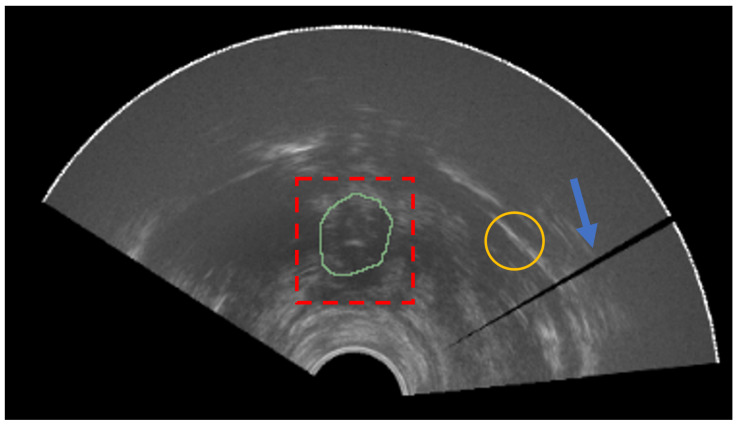
Prostate apex (ground-truth mask in green) is not easily distinguishable from the rest of the image (red dashed box). The yellow circle represents an example of a region with low signal-to-noise ratio. The blue arrow denotes a shadow artifact.

Prostate cancer is a major health problem and represents the most common cancer in the male population, accounting for 18.5% of all the cancers diagnosed in humans [1]. The number of new cases worldwide passed 1,275,000 and caused approximately 360,000 deaths in 2018 alone (3.8% of all deaths caused by cancer in men) [2]. Numerous imaging modalities are employed for prostate cancer diagnosis, treatment, and follow up. Transrectal ultrasound (TRUS), magnetic resonance imaging (MRI), and computed tomography (CT) are the most common employed imaging modalities [3]. Each technique provides different information and is used for several divergent clinical scopes. During biopsy procedures, TRUS is commonly employed since it is an inexpensive, portable, and real-time methodology [4]. MRI is mainly adopted for diagnosis and treatment planning [5]. In fact, this modality has better soft tissue contrast and allows more efficient lesion detection and staging in patients affected by prostate cancer. As can be seen from Figure 1, the TRUS images suffer from problems such as speckle, low contrast, and shadow artifacts [6]. Calcification and acoustic shadowing make the automatic segmentation of prostate region a very complex task [7]. The prostate usually appears like a hypoechoic mass encompassed by a hyperechoic region [8]. CT scans are useful in determining if prostate cancer has spread to bone tissues or to assess the effectiveness of the brachytherapy [9,10].

In clinical practice, the majority of prostate cancer cases are diagnosed prior to symptoms development, thanks to the prostate-specific antigen (PSA) [11] levels in the blood and rectal examination. In order to achieve detailed information, MRI is the preferred modality, with PI-RADS v2.1 being the standard for finding interpretation [12].

The standard prostate biopsy procedure involves the extraction of 10–12 tissue samples. Since there is no guarantee that sampling prostate in these regions is the most effective way to obtain the regions with cancerous tissue, fusion-guided prostate biopsy is now becoming the preferred modality for most urologists and surgeons. In this way, suspicious areas found in the MRI of the prostate can be targeted during the prostate procedure exploiting the fusion with real-time TRUS imaging, also allowing a better view of the biopsy needle. The advantages of this approach include the following: more accurate sampling of the cancerous tissue in the prostate gland; less patient tissue is extracted; less pain; less risks for the patient, including faster recovery time [13].

In order to implement a fusion-guided prostate biopsy framework, segmentation of the prostate gland must be obtained from both TRUS and MRI domains. Exceptions involve systems in which images are manually registered by the user at procedure time, by superimposing MRI over the TRUS. Since MRI is acquired days before the prostate biopsy, it is not fundamental that its segmentation is performed in real time. Th manual segmentation of prostate from MRI is a tedious task and prone to inter- and intra-radiologist variation [14], so the exploitation of an automatic method grounded on the nnU-Net framework [15] can further ease the procedure and improve its diagnostic accuracy. It is worth noting that nnU-Net does not denote a novel network topology, loss function, or training procedure. Indeed, nnU-Net stands for “no new net”. The strength of the nnU-Net framework comes from the systematization of all the steps which were usually manually tuned in the training pipeline of semantic segmentation architectures, including data augmentation, hyperparameters’ tuning, test-time augmentation, and ensembling. Instead, manual segmentation of the prostate gland from TRUS has to be realized real time during the prostate procedure; therefore, the need for a fast and effective methodology for this task is crucial in clinical practice.

Ghose et al. performed a comprehensive survey which focused on methods for prostate segmentation in TRUS, MR, and CT images [3]. Prostate gland segmentation eases multimodal image fusion for tumor localization in biopsy. Manual annotation of radiological images is a tedious and error-prone task, which also has problems of inter- and intra-radiologist variability. Fully automatic methods, such as those based on deep learning (DL), require huge annotated data, usually for the same transducer that will be used for the procedures, since there is a high variability in ultrasound image quality across vendors. Nonetheless, when data are available, DL methodologies show their strength, as is the case for deep attentional features (DAF) [16] and DAF 3D [17]. The shortcoming of these techniques is that they cannot be applied before having acquired a dataset with images of the same ultrasound device that will be adopted during procedures.

Mahdavi et al. [18] proposed a semiautomatic prostate segmentation method that can be applied in prostate brachytherapy setups. The 3D geometric model of the prostate is created based on prior knowledge of the shape of the gland and on the assumption that the prostate has a tapered ellipsoidal shape and is slightly warped posteriorly due to the presence of the TRUS probe. They used a tapered and warped ellipsoid as the prior shape of the prostate gland. The proposed segmentation algorithm requires a manual initialization of the physician: on the mid-gland image, the user selects six boundary points following a specific criterion. The main disadvantage of this method is that it requires the user to input initialization points in a very precise way, and relies deeply on these points, posing problems if they are slightly inaccurately placed or when the prostate region has an irregular shape.

Gong et al. [19] incorporated deformable superellipses in a Bayesian segmentation framework, exploiting an edge-detection algorithm for discovering prostate boundaries. They show the capacity of deformable superellipses to capture the prostate shape in various anatomical zones. The main limitation of this method is that it requires an initial contour that is similar to the real boundaries of the prostate gland. To overcome this issue, Saroul et al. [20] proposed a variational approach, exploiting the implicit representation of a superellipse for modeling the active contour.

This work addresses the problem of providing a fast and reliable semiautomatic method for prostate segmentation from TRUS images, that can be employed without having acquired a specific dataset for the transducer in consideration. The approach, like those mentioned before, is based on the theory of deformable superellipses. Two kinds of methodologies can be employed for achieving segmentation with superellipses. In the first, image characteristics, as edge maps or region energy, are employed for performing automatic image segmentation [19,20]. In the second case, geometry of the prostate is inferred from user-defined points [18].

The method of Gong et al. [19] requires the generation of edge maps in order to produce the final segmentation, whereas the approach of Saroul et al. [20] needs assumptions to be made about region energy. On the other hand, the proposed approach does not need to make these assumptions on input data, given that image quality can vary widely among different transducers.

Compared with the approach of Mahdavi et al. [18], the proposed method does not require to select points in a refined geometric way, but the user has flexibility on how to place them. Moreover, the proposed formulation allows the modeling of more irregular 3D shapes, since the user can insert points in an arbitrary number of slices, thus eventually obtaining shapes which go beyond tapered and warped ellipsoids.

Extensive experiments have been carried out to outline the best guidelines that a human operator should take into consideration when using the proposed algorithm for performing a procedure in order to minimize the number of points required and maximize the segmentation accuracy. In any case, performing a second iteration can mitigate eventual problems that arise after non-optimal points are placed in the first iteration.

Lastly, an application of the proposed method in an image fusion setup with MRI is shown. The segmentation module for the MRI relies on the nnU-Net framework [15]. Segmentation masks from both domains are then registered in order to accomplish the image fusion task.

The remainder of the paper is structured as follows: Section 2 describes the materials and methods considered for this study, including the dataset and the developed methodologies for segmentation from MRI and TRUS, and the setup of MRI–TRUS registration. Thereafter, Section 3 presents the results for both the segmentation and the registration tasks. Section 4 discusses the obtained results, and Section 5 concludes the paper and offers future perspectives.

## 2. Materials and Methods

### 2.1. Dataset

A dataset containing anonymized imaging data of human prostate of *N* = 3 patients was made publicly available by Fedorov et al. [21]. This dataset will be referred to as ZENODO throughout this paper. For each patient, both MRI and TRUS examinations were performed. The former was performed with the purpose of staging the disease and the latter was performed with the aim of completing a volumetric examination to prepare the brachytherapy implant. Both modalities are 3D scalar images. Annotations provided by Fedorov et al. include manual segmentation of the whole prostate gland for both MRI and TRUS, and fiducial points placed in specific anatomical sites to improve subsequent image registration and fusion. In particular, fiducials are placed at the urethra entry into the prostate at base (UB), the verumontanum (VM), and the urethra entry into the prostate at apex (UA).

In order to validate DL models for the task of MRI prostate segmentation, the datasets PROMISE12 [14] and SAML [22,23] were also included in the analysis.

The ZENODO dataset was employed to test the proposed method for TRUS segmentation, MRI segmentation, and TRUS–MRI registration, whereas PROMISE12 and SAML were employed to validate the nnU-Net model for MRI segmentation.

**Figure 2:**
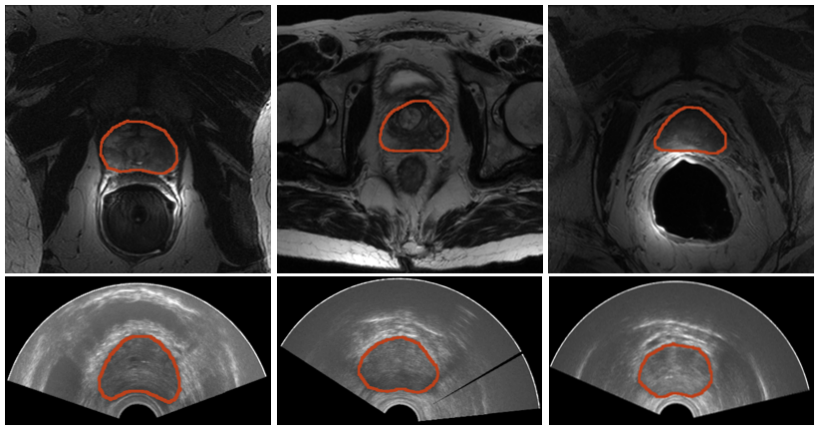
Samples of images from both modalities. (**Top**) Prostate MRI. From left to right, a sample image for each of the datasets—PROMISE12, SAML, and ZENODO—is shown. (**Bottom**) Three sample prostate TRUS from the ZENODO dataset.

Sample images for both domains, TRUS and MRI, are reported in Figure 2. A summarized table for the considered materials is provided in Table 1.

**Table 1:** Datasets description. As imaging modalities, only ZENODO contains TRUS images, even in a very limited quantity, being only 3. Fiducial points are also present only in the last dataset. Seg—segmentation; Reg—registration.

Dataset	Imaging Modality	Task	Number of Images	Ground Truth Segmentation	Fiducial Points	File Format
PROMISE12 [14]	MR (T2W)	MR Seg	50	✓	**✗**	NIfTI
SAML [22,23]	MR (T2W)	MR Seg	116	✓	**✗**	NIfTI
ZENODO [21]	MR (T2W), TRUS	TRUS Seg, MR/TRUS Reg	3, 3	✓	✓	NRRD

### 2.2. Workflow

**Figure 3:**
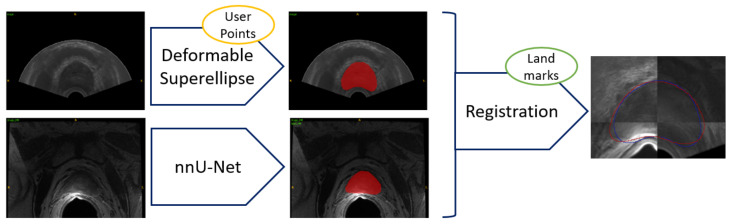
Workflow for TRUS and MRI segmentation and subsequent image fusion. Segmentation from TRUS is achieved in a semiautomatic way by fitting a 3D model based on deformable superellipses starting from user-defined points in at least three slices. Segmentation from MRI is performed fully automatically by exploiting the nnU-Net framework. Registration can be either performed in an automatic way, or the user can add anatomical landmarks to constrain the space of transformations.

The workflow employed for achieving image fusion, starting with segmentation for both imaging modalities, namely TRUS and MRI, is reported in Figure 3. In the clinical practice, segmentation does not happen at the same time, since MRI segmentation can be achieved preoperatively, whereas the TRUS segmentation has to be obtained intraoperatively, at the start of the prostate biopsy procedure.

Segmentation from MRI involves a preprocessing stage, so that images can be fed to a deep learning architecture, the nnU-Net. Lastly, a postprocessing is performed, so that noisy elements can be removed from images (e.g., only one connected component is expected), increasing segmentation accuracy. The described operations can be carried out in a fully automatic way. MR images are especially important for identifying target region for biopsy, since they have better contrast than other imaging modalities. Details are described in Section 2.3.

Segmentation from TRUS is achieved with a semiautomatic algorithm, which requires input points from the user. The physician has to annotate points in at least three slices of the prostate gland in axial planes, taking care when placing points in the deformed zones (the transducer itself introduces deformation). Starting from this point, a deformable superellipse is fitted with an optimization algorithm. Then, interpolation is employed to achieve 3D reconstruction of the prostate gland. The entire procedure is explained in Section 2.4.

Then, having both segmentation masks from TRUS and MRI modalities, registration can be performed, enabling image fusion, which allows tissue coming from both modalities to be seen at the same time. Optionally, a set of anatomical landmarks can be inserted by the user to ease and constrain the registration optimization step. The procedure is presented in Section 2.5.

### 2.3. MRI Segmentation

The semantic segmentation of the prostate gland from MRI can be efficiently met via DL techniques, as fully convolutional neural networks [24]. Semantic segmentation, which poses the basis for subsequent classification and characterization tasks [25,26], is essential in numerous clinical applications including artificial intelligence in diagnostic support systems, therapy planning, intraoperative assistance, and monitoring of tumor growth. It is a computer vision task that can be computed with DL algorithms and consists of labeling each pixel of an input image, without recognizing the different instances of objects [27,28]; it is possible to see semantic segmentation as a problem of conversion from image to image, where the input image is the original image and each pixel intensity value of the output image indicates the relation of that pixel to the associated class [29].

Most semantic segmentation architectures are based on encoder–decoder networks. The encoder is devoted into the process of feature extraction or subsampling. Decoding is an upsampling operation, in which the spatial information output from the encoding layer is reconstructed, increasing the spatial resolution. The encoder–decoder structures have been implemented in different convolutional network architectures, including SegNet [30], U-Net [31], U-Net 3D [32], and V-Net [33]. Besides prostate segmentation, applications in medical imaging tasks of those architectures encompass liver vessels delineation [34], segments classification [35], lung COVID-19 lesions segmentation [36], and vertebrae segmentation [37].

In this work, in order to perform the semantic segmentation of the prostate gland from MRI, the nnU-Net framework has been exploited. It allows the semantic segmentation tasks to be approached with standardized pipelines [15,38], and its architecture is based on those of U-Net and U-Net 3D. It was originally conceived during the Medical Decathlon Segmentation Challenge [39], where it emerged as the leading approach in all tasks. Advantages of this method consist of automatic configurations of preprocessing, data augmentation, training, inference, and postprocessing. Parameters to set for training nnU-Net include number of epochs, initial learning rate, batch size, patch size, and the sum of dice loss and cross-entropy as a loss function.

### 2.4. TRUS Segmentation

Segmentation of anatomical structures in noisy data, such as TRUS images, is a complex task since boundaries are not clearly defined, as shown in Figure 1. Therefore, the adoption of a prior information about the geometric structure of interest is useful to constrain the model deformation [40,41]. Deformable models can be used to achieve this result.

Geometry, physics, and mathematical optimization lay the foundations for the segmentation algorithms based on deformable models [40]. The constraint on the model shape is derived from geometry, where the evolution of the shape in space is governed by physical theories, whereas in order to fit the model to the accessible data, the optimization theory is employed [42]. Segmentation of anatomical structures in deformable models is achieved by exploiting an energy minimization framework. Two kinds of energies are considered: internal and external energies. The deformable model is propagated in the direction of the object contours by external energies, whereas the smoothness of the boundaries are preserved by internal energies. The deformable model framework comprises of various methodologies, that, according to Ghose et al. [3], can be categorized into deformable mesh, active-shape models, level sets, active-contour models, and curve-fitting models. More advanced approaches may include a mixture of these techniques, with the idea that merging information concerning boundaries known a priori—the region, shape, and features of the prostate region—can provide more accurate models, such as the deformable superellipse formulation of Gong et al. [19].

#### 2.4.1. Shape Models

In a wide variety of medical imaging scenarios, the general location, orientation, and shape of the object of interest are known a priori. As reported by previous studies concerning TRUS images, prostate contours appear smooth and with a closed, near-convex shape [19]. This information can be embedded into the deformable model in different forms: as initial conditions, as a way of constraining model shape parameters, and as a model fitting procedure. Global shape properties can me modeled with parametric shape models. The advantage of this technique is that it does not require the presence of anatomical landmarks.

Furthermore, representation of the shapes can be tackled with many different methods [43,44]. For instance, Tutar et al. [45] proposed that the 3D prostate boundaries could be modeled with spherical harmonics of degree eight. Local deformations can be controlled, thanks to the exploitation of parameters, leading to the capacity of modeling complex shapes. Additionally, there is an increment in the computational complexity.

Similarly, reducing the number and range of parameters can allow the global shape to be modeled in approaches which are stable and quick from a numerical perspective, leading to more compact representations. In the following section, the authors introduce the deformable superellipse, a powerful model for the geometry of the prostate gland [19]. When the deformable superellipse is not capable of properly modelling all the nuances of the prostate region in a 2D slice, bidimensional B-splines [46] can be employed in the proposed approach, obtaining very refined results while retaining the possibility of modelling a 3D shape with a relatively low number of parameters.

#### 2.4.2. Deformable Superellipses

**Figure 4:**
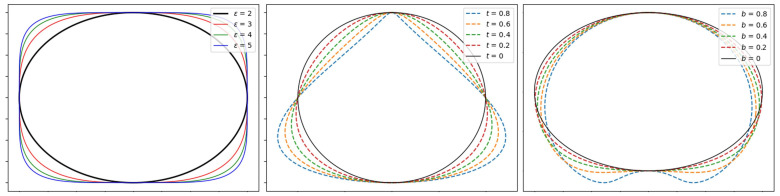
Deformable superellipse modeling capabilities examples. The left image represents the superellipse varying squareness ϵ parameter, whereas the middle one depicts the deformable superellipse varying tapering *t* parameter, and the right one portrays the deformable superellipse varying circular bending *b* parameter.

Superellipses allow ellipses to be generalized in a natural way. Different base geometrical shapes can be modeled through superellipses, as ellipses, parallelograms, rectangles, and pinched diamonds by handling a small number of parameters [47,48]. Examples of shapes that can be modeled by superellipses are portrayed in Figure 4. The 3D generalization of the superellipse, the superellipsoid, has not been considered since it makes assumptions about the 3D regularity of the prostate shape which are too simplistic.

A centered superellipse can be expressed in the following **parametric** form, reported in Equation (1):

(1) $$\left\{\begin{array}{c} x=a_{x}\cdot {\left|cos\left(\theta \right)\right|}^{\frac{2}{\epsilon }}\cdot sign\left(cos\left(\theta \right)\right)\\ y=a_{y}\cdot {\left|sin\left(\theta \right)\right|}^{\frac{2}{\epsilon }}\cdot sign\left(sin\left(\theta \right)\right) \end{array}\right.$$

where the size parameters $a_{x}>0$, $a_{y}>0$ define the length of the semi axes, and ϵ>0 specifies the squareness in 2D plane, as shown in Figure 4. The corresponding **implicit** form is given by the Equation (2):

(2) $${\left|\frac{x}{a_{x}}\right|}^{\epsilon }+{\left|\frac{y}{a_{y}}\right|}^{\epsilon }=1$$

The *inside*–*outside* function is reported in Equation (3):

(3) $$f\left(x,y\right)={\left|\frac{x}{a_{x}}\right|}^{\epsilon }+{\left|\frac{y}{a_{y}}\right|}^{\epsilon }$$

where if f(x,y)=1, then the point (x,y) lies on the superellipse; if f(x,y)>1, then the point (x,y) lies outside the superellipse; if f(x,y)<1, then the point (x,y) lies inside the superellipse.

The superellipse model does not allow, in this version, all deformations which are required to build a proper model of the prostate gland to be obtained. Nonetheless, geometric deformations can result in a variety of shapes which are modeled by deformable superellipse, as translation, rotation, tapering, and bending [49,50]. Moreover, these transformations can be modeled with a few number of parameters, given that translation with respect to an axis, rotation, tapering, and bending are described each with a single parameter. Deformable superellipses can then be characterized by a parameter vector p, defined as in Equation (4):

(4) $$p=\{a_{x},a_{y},l_{x},l_{y},r,\epsilon ,t,b\}$$

where ϵ is the squareness parameter and $a_{x}$, $a_{y}$ are the semi-axes lengths defined above. Other parameters are those involved in the global similarity transformations for superellipses [19]: $l_{x}$, $l_{y}$ are the translations along *x* and *y* axes, *r* is the rotation angle, and *t* and *b* the tapering and circular bending on the *y*-axis, respectively.

Details of all these geometric transformations are reported in Appendix A, whereas inverse transformations are reported in Appendix B. Example of deformable superellipse modeled by variations in tapering and bending are reported in Figure 4.

##### Optimization Framework

In the Bayesian framework proposed in Gong et al. [19], the authors assumed that some parameters (those concerning shape) have a Gaussian distribution as prior, $N(p_{s})$, whereas others (those concerning pose) have a uniform distribution as before, $U(p_{p})$. The edge strength likelihood is denoted as *E*. Then, according to the Bayes rule, the posterior probability can be modeled as in Equation (5):

(5) $$Pr\left(p\;|\;E\right)=\frac{Pr(p)\cdot Pr(E\;|\;p)}{Pr(E)}=\frac{Un\left(p_{p}\right)\cdot N\left(p_{s}\right)\cdot Pr\left(E\;|\;p\right)}{Pr(E)}\propto Un\left(p_{p}\right)\cdot N\left(p_{s}\right)\cdot Pr\left(E\;|\;p\right)$$

This results in optimizing the log likelihood in Equation (6):

(6) $$L=ln\left(Pr\left(p_{s}\right)\right)+ln\left(Pr\left(E|p\right)\right)$$

#### 2.4.3. Proposed Approach

In the proposed approach, the deformable superellipse is modeled as specified in Section 2.4.2. Geometric deformations to the base superellipse shape can be obtained as reported in Appendix A.

The problem of modeling Pr(p | E), as in Equation (5), is that it requires prior data on edge maps from images of the same kind of the ultrasound device that will be used for carrying out the procedures. When it is not feasible to collect such images in advance, it may be preferable to model Pr(p | U), where *U* is a set of user-defined points. If the model does not require rigid assumptions about *U*, it can provide a fast and reliable system for achieving prostate gland segmentation with only moderate user interaction and without the need to build a large training set.

Therefore, in the proposed formulation, the posterior probability can be written, as reported in Equation (7):

(7) $$Pr\left(p\;|\;U\right)=\frac{Pr(p)\cdot Pr(U\;|\;p)}{Pr(U)}\propto Un\left(p_{p}\right)\cdot N\left(p_{s}\right)\cdot Pr\left(U\;|\;p\right)$$

The prior about shape parameters can be optimized by maximizing Equation (8) [19]:

(8) $$ln\left(Pr\left(p_{s}\right)\right)=-\sum_{j}\left[\frac{{\left(p_{j}-m_{j}\right)}^{2}}{2\cdot \sigma_{j}^{2}}\right]$$

Instead, the likelihood linked to the term Pr(U | p) can be maximized by optimizing the energy in Equation (9), where *U* is the set of user-defined points, *C* is the polygon representing the prostate mask boundaries, *d* is the point to polygon distance, and E(C;U) is the energy function to minimize.

(9) $$E\left(C;U\right)=\sum_{(x,y)\in U}d{\left(C,(x,y)\right)}^{2}$$

Afterwards, 2D superellipses were fit to slices where users insert points, a 3D model can be reconstructed with the performance of linear interpolation of the parameters involved in the vector **p**. In order to build a 3D model of the prostate gland, a minimum of three slices have to be labeled. The annotated slices must include base, apex, and mid-gland regions of the prostate gland. On the base and the apex, a minimum of 4 points must be inserted by the user, whereas on the mid-gland, a minimum of 6 is recommended. For mid-glands which have irregular shapes, a number of points up to 12 may be beneficial.

Since the user can add more than three slices, shapes which are more complex than one tapered and warped superellipsoid or two semi-superellipsoids can be obtained. The following paragraph describes how the optimization algorithm is carried out during a procedure with an operator.

#### 2.4.4. Implementation Details

**Figure 5:**
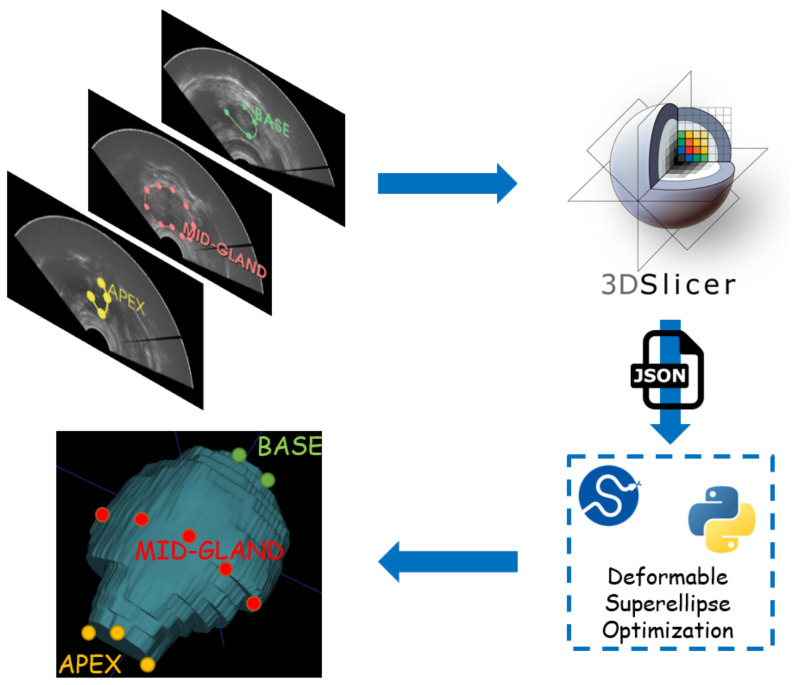
Workflow for TRUS segmentation with the developed methodology. Physicians must annotate some points in the apex, the base, and the mid-gland of the prostate. Then, a JSON file is fed as input to an optimization routine which fits the best 2D superellipse in every annotated slice. Then, a 3D model is built by linearly interpolating 2D models. 3D Slicer has been employed as graphical interface to speed up and ease the process.

The general workflow employed for TRUS segmentation is reported in Figure 5.

First, the user is asked to select points from at least three slices of the TRUS volume. In every slice, the user has to select a variety of points ranging approximately from 4 to 12, as detailed at the end of Section 2.4.3. In order to ease this process for the experiments realized during this research, the authors created a JSON interface with the popular 3D Slicer software [51].

The user can enter two types of models when inserting points. The first is the superellipse, and the second exploits bidimensional B-splines (as implemented by the method scipy.interpolate.splprep). For the purposes of 3D modeling, a superellipse is then fitted to the spline in the second case. For mid-gland slices, the B-spline configuration, especially when 10–12 points are annotated by the user, is the recommended way to proceed. When there are few annotated points, a deformable superellipse is more likely to properly work, considering that it has a relatively low number of parameters. In particular, in the configuration with the least possible number of points, where the user places 4 points at base, 6 points at mid-gland, and 4 points at apex, the deformable superellipse should be exploited.

In order to effectively implement the optimization procedure of the 2D superellipse to the slice points, an iterative minimization procedure has been carried out. At every iteration, the optimizer passes a vector p of parameters to a superellipse class, which builds an object with the given parameters and measures its energy with respect to the user-defined points.

After the object is created, the *inside*–*outside* function reported in Equation (3) is used to create a mask of points which satisfies the condition for the centered superellipse. Then, these points are transformed by using deformations in the following order: rotation, as defined in Equation (A2); linear tapering along *y*-axis, as defined in Equation (A3); circular bending along *y*-axis, as defined in Equation (A4); translation, as defined in Equation (A1).

The mask obtained by these transformations is subject to morphological closing operator, since holes arise during the transformation process. Then, the energy for the built superellipse can be defined as the sum of distances from user-defined points to the polygon of the mask boundary. The point-to-polygon distance can be calculated with the pointPolygonTest method from the OpenCV library. At the end of the minimization procedure, the optimizer will find the best vector p for the input points given by the user.

Lastly, the 2D deformable superellipse models fit in multiple slices (at least three including the base, the apex, and the mid-gland) which are employed to reconstruct the 3D volume by performing linear interpolation of the parameters contained in the vector p. The program also returns a list of JSON files which can be loaded in 3D Slicer to refine the segmentation results and eventually perform a second iteration. In the second user iteration, B-splines are employed for providing the contour of the prostate gland, since the user only need to adjust boundary points provided by the previous iteration of the algorithm.

### 2.5. MRI–TRUS Registration

The described registration algorithm is segmentation-based, so that both MRI and TRUS segmentation masks are required for performing the procedure. Other authors have also considered this step to be fundamental [52,53]. The particular challenge of MRI–TRUS registration is that the anatomical areas visible in one modality may not be visible in the other.

Before starting with the registration procedure, preprocessing is performed with the purpose of improving and easing the fusion algorithm results.

First, the 3D images are cropped into 3D bounding boxes (i.e., volume of interest (VOI)) that extend 10 mm over the margin delineated by the segmentation mask. Then, the VOIs are resampled to make them isotropic with the same resolution for both modalities. For the binary segmentation mask, nearest neighbor interpolator is employed to perform the resampling. Output resolution is set to 0.3 mm × 0.3 mm × 0.3 mm.

**Figure 6:**
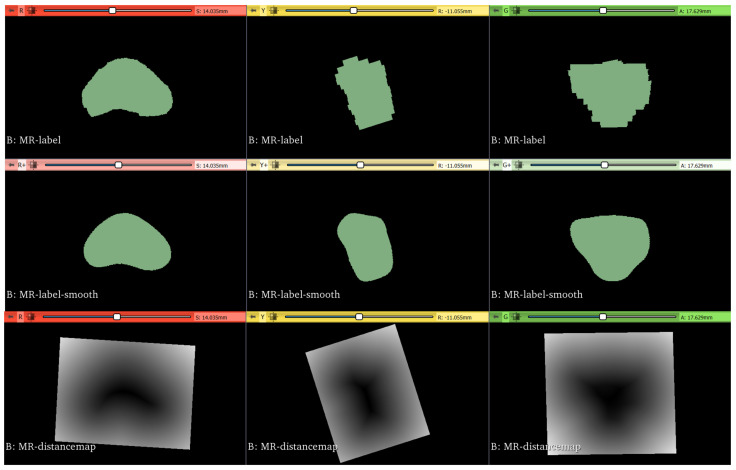
Original ground-truth segmentation masks are reported in the top row. Then, they are smoothed with a Gaussian filter, as depicted in the middle row. Lastly, distance maps are obtained from the smoothed masks, as shown in the bottom row.

Segmentation masks are smoothed with a Gaussian kernel with σ=3 [53]. Lastly, the Maurer signed-distance transformation, which exploits Euclidean distance transform [54], is applied to the segmentation masks. The steps involved in the registration preprocessing are reported in Figure 6.

The purpose of the initialization is to simplify the calculation of the center of rotation and translation needed for the rigid transformation. Two kinds of initialization have been considered: (i) based on the center of images; (ii) based on a set of landmarks.

In the first case, the centers of images are calculated in the coordinate spatial system, considering the origin, dimensions, and spacing of images. The geometric center of the moving image is given as initial center of the rigid transformation, and the vector that goes from the center of the fixed image to the center of the moving image is given as the initial translation vector.

The second approach, instead, determines an initial transformation by considering a set of landmarks. It determines the optimal transform that can map the fixed image and the moving image with respect to least-square errors of the levels of intensity [55].

**Figure 7:**
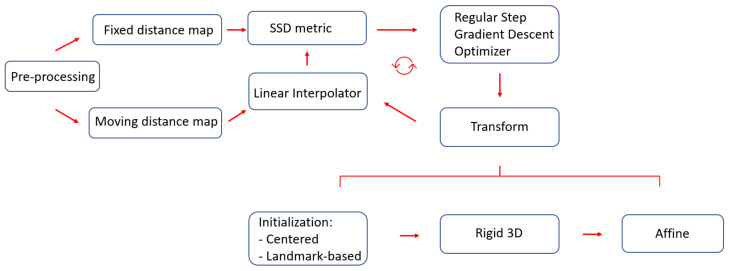
The proposed registration workflow starts with preprocessing segmentation masks, to make them isotropic with the same resolution. Thereafter, SSD is employed as a metric to perform the registration, where gradient descent is adopted as the optimizer. Two kinds of initialization were considered: one based on centers and the other based on landmarks.

Since the proposed approach aims to perform the registration of distance maps whose intensity values have the same range of values and meaning, the dissimilarity measure employed is the sum of squares of intensity differences (SSD). Lower values of the said metric correspond to better results. The optimizer employed is based on gradient descent, whose aim is to find the set of parameters which define a transformation that optimize the metric as well as possible. The overall workflow employed for the registration with all the various components described in this section is portrayed in Figure 7.

### 2.6. Performance Metrics

The performance of the segmentation and registration algorithms analyzed for this study was evaluated by calculating metrics based on the overlap of volumes and metrics based on the distances of the external surfaces points. The metrics used for volumetric overlap require the predicted volume, *P*, and the ground-truth volume, *G*, to be introduced. They were the *dice similarity coefficient* (DSC), the *volumetric overlap error* (VOE), and the *relative volume difference* (RVD), which are defined as in Equations (10)–(12), respectively.

(10) $$DSC\left(P,G\right)=\frac{2\cdot |P|\cap |G|}{\left|P|+|G\right|}$$

(11) $$VOE\left(P,G\right)=1-\frac{\left|P\cap G\right|}{\left|P\cup G\right|}$$

(12) $$RVD\left(P,G\right)=\frac{\left|P|-|G\right|}{\left|G\right|}$$

The metrics based on the concept of surface distances include the *Hausdorff distance* (HD) and the *average symmetric surface distance* (ASSD). Definitions for these metrics can be found in [24].

## 3. Results

### 3.1. Segmentation

#### 3.1.1. MRI

**Figure 8:**
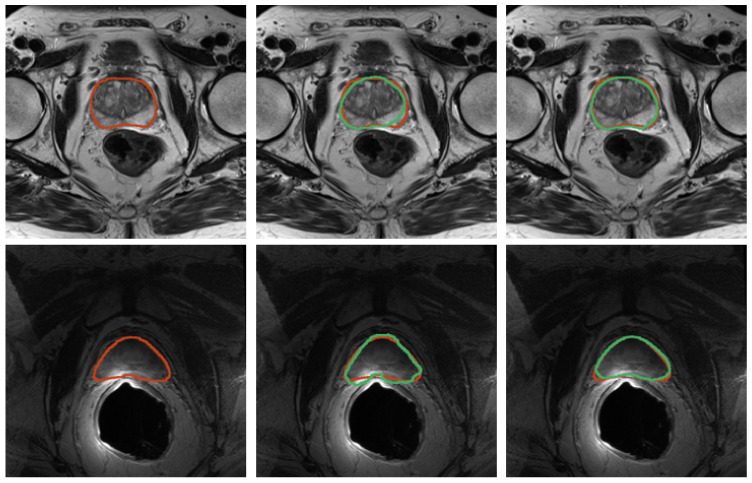
Results for prostate segmentation from MRI. Top row contains images from the SAML dataset and the second row contains images from ZENODO. The ground truth is represented in red, whereas the predictions from the nnU-Net models are colored in green. The middle image shows the prediction mask for the nnU-Net trained for only 10 epochs, whereas the right image depicts the prediction mask for the one trained on the PROMISE12 dataset.

Quantitative results for MRI segmentation with nnU-Net are reported in Table 2, and the qualitative results, as segmentation masks, are depicted in Figure 8. The nnU-Net model trained on the PROMISE12 challenge obtained the best results [14,15]. The SAML-V dataset was obtained by sampling 24 images for validation from the SAML dataset. It is worth noting that the Dice coefficient is higher than 88% and ASSD is less than 1 mm for both validation sets under consideration, showing the reliability of the nnU-Net framework for the automatic MRI segmentation of the prostate region.

#### 3.1.2. TRUS

**Figure 9:**
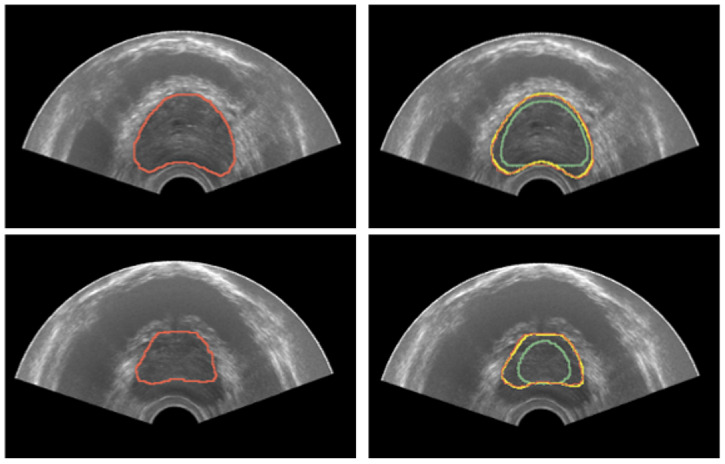
Results for prostate segmentation from TRUS. The left image portrays the ground-truth prostate mask in red. The right image depicts the segmentation results after both the first and second iteration in green and yellow colors, respectively.

Quantitative results for TRUS segmentation with the developed methodology based on deformable superellipses are reported in Table 3, whereas sample segmentation images are depicted in Figure 9. Three experiments have been conducted per each case, placing 4 points on the base and 4 on the apex, using only the superellipse to fit the contours. Instead, on the mid-gland, a number of points varying from 10 to 12 has been considered, exploiting B-splines before fitting the superellipse to finally achieve the 3D modeling of the prostate gland. Results are reported as mean ± std of the experiments performed on each case. It is possible to see that results are considerable overall, with the Dice coefficient being greater than 87% in all cases. Moreover, the proposed implementation is iterative, so that the user can refine the results until it reaches the desired performance. For the purposes of this research, the experiments stopped at second iteration, which allowed the results to be improved in all cases.

### 3.2. Registration

**Figure 10:**
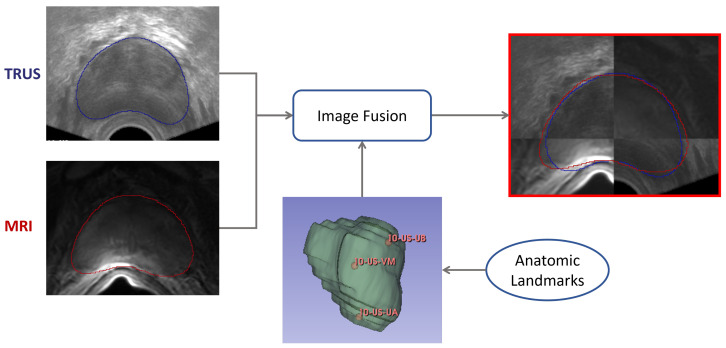
Workflow employed for the image-fusion procedure. Segmentation masks are obtained for both domains—TRUS and imaging. Then, the registration is performed as described in Section 2.5, so that images can be fused. Both masks are shown after the registration procedure.

Quantitative results for registration across the two considered imaging modalities, TRUS and MRI, are reported in Table 4 for the configurations with and without landmarks, respectively. An example of the workflow for the image fusion is depicted in Figure 10. Dice Coefficient is higher than 91% for all the cases, and HD is less than 4 mm, demonstrating that the developed registration method is promising.

## 4. Discussion

Prostate segmentation is a pivotal but strenuous-to-accomplish task which is required for targeted prostate biopsy procedures. Moreover, every transducer for TRUS can produce different images, resulting in a variety of conditions which make it difficult to transfer what has been learned on one dataset to another. Lastly, the lack of annotated data for TRUS segmentation, from the ZENODO dataset—that consists of only three images, and is the only dataset available for this research—adds to the peculiarity to the task.

The deformable superellipses are shape models that allow a variety of geometry deformations to be modeled starting from ellipses, which can resemble most common prostate shapes. In fact, the prostate shape is characterized by being a tapered ellipsoid [18]. When the procedure is performed, the transducer induces a slightly posterior deformation in the patient’s prostate which can be modeled, for instance, with the bending parameter, *b*.

Therefore, this work proposed a novel formulation of the deformable superellipse to make it a suitable method for TRUS segmentation, even in the absence of training data from a given transducer. Other approaches, such as that of Gong et al. [19], require edge-detection algorithms; therefore, that approach could be employed for automatic segmentation. However, such an approach requires training data from a specific transducer. The advantage of the proposed method is that it can be applied in any circumstance, only requires a moderate interaction with a physician, and always yields considerable results.

In the experiments carried out for this study, the proposed method requires 41 ± 7 s for placing the points in three or four slices, whereas it takes 5 ± 1 s to build the 3D model. The time needed for the second iteration is more variable—74 ± 32 s. The superellipse implementation of Mahdavi et al. [18] took 32 ± 14 s for initialization, which is similar to the time needed to place the initial user-defined points in the proposed approach. On the computational side, it needed 14 ± 1 s, which is more than the considered implementation. Furthermore, in their case, segmentation refinement can be performed by the user, with a time ranging between 1 and 3 min. It is not possible to directly compare the proposed approach with the work of Gong et al. [19], since their method is capable of performing segmentation in less than 5 s per slice, but it only delineates 2D boundaries.

The developed methodology managed to achieve decent results, reaching a Dice coefficient greater than 87% in all the images considered for the test, coming from the ZENODO dataset. Then, the research focused on proving the applicability of this module in a targeted biopsy setup. So, the nnU-Net framework was employed for the task of performing segmentation from MRI, achieving a Dice coefficient higher than 88% on the SAML-V dataset and higher than 91% on the ZENODO dataset.

Lastly, the authors developed a custom registration procedure, which allowed a Dice coefficient higher than 91% to be reached in all cases, and an HD lower than 4 mm to be reached in all cases, showing the effectiveness of the proposed framework in clinical applications. In the registration framework, both an initializer based on centers of images, and another which relied upon a set of landmarks, were considered. From the obtained results, it is possible to note that the former allowed Dice coefficients of 91.77%, 94.82%, and 93.61% to be reached, and HD of 3.77 mm, 2.12 mm, and 3.55 mm to be reached. The latter achieved Dice coefficients of 91.78%, 94.85%, and 93.60%, and HD of 3.77 mm, 2.09 mm, and 2.29 mm. Hence, the two methods provides similar results; therefore, eventually, a simpler center-based initialization can be adopted for the affine registration procedure.

Overall, the obtained results for both the segmentation methods are satisfactory for implementation in a targeted prostate biopsy setup. The registration framework can eventually be improved, in order to accommodate deformable prostate biopsy setups, which will allow even better results for the image-fusion steps.

## 5. Conclusions

Prostate segmentation from MRI and TRUS is a complex challenge, but is very useful in clinical setups. With MRI, with the advent of the nnU-Net framework, the challenge is more easily met, since a standardized pipeline can be employed for semantic segmentation. However, there is still a lack of substantial data and standardized methodologies for TRUS images. In this work, the authors proposed an approach that can be employed in the absence of training data; this approach only relies on the theory of deformable superellipses. With the only requirement of a moderate interaction with the user, the developed methodology reliably segments the prostate from TRUS images. In order to show the effectiveness of the overall workflow, as well as in the clinical setups, an image-fusion procedure which relies on image registration between TRUS and MRI was developed. Thus, we have successfully realized a semiautonomous segmentation framework for prostate cancer from TRUS images, without relying on a large-scale dataset. Furthermore, the proposed framework can be employed as an annotation tool to ease and speed up the construction of prostate segmentation datasets, so that eventually fully automated methods can be developed. In future works, deformable registration techniques can be considered to further improve the image-fusion step.

## Figures and Tables

**Table 2:** Quantitative metrics results for MRI prostate segmentation with nnU-Net. Performance has been measured on two validation sets.

Train Set	Epochs	Test Set	Dice [%]	*RVD* [%]	HD [mm]	ASSD [mm]
PROMISE12	1000	SAML-V	88.18 ± 10.53	17.58 ± 31.61	21.03 ± 51.06	0.86 ± 1.14
PROMISE12	1000	ZENODO	91.17 ± 1.19	4.13 ± 8.79	16.11 ± 3.56	0.26 ± 0.01

**Table 3:** Quantitative metrics results for TRUS prostate segmentation with the proposed superellipse-based approach. Results are reported for both the first and second iterations of the algorithm run.

Metrics		Dice [%]	*RVD* [%]	HD [mm]	ASSD [mm]
**Case 9**	1st	87.15 ± 2.41	−13.27 ± 8.34	25.12 ± 5.58	0.53 ± 0.120
	2nd	88.56 ± 2.66	−9.44 ± 8.88	16.10 ± 7.12	0.38 ± 0.022
**Case 10**	1st	89.31 ± 1.13	−12.21 ± 3.06	9.25 ± 2.41	0.23 ± 0.020
	2nd	92.57 ± 0.45	−4.86 ± 0.36	9.37 ± 2.53	0.17 ± 0.015
**Case 12**	1st	90.76 ± 1.39	−5.46 ± 3.61	23.30 ± 9.58	0.30 ± 0.049
	2nd	92.47 ± 0.30	−1.87 ± 1.24	21.26 ± 8.22	0.23 ± 0.048

**Table 4:** Registration results in both configurations. The first utilizes the center of the images as the initializer, and the second utilizes a set of landmarks as the initializer.

Experiments	Dice [%]	Jaccard [%]	*RVD* [%]	HD [mm]
case 10—center	91.77	84.79	−0.86	3.77
case 10—landmarks	91.78	84.80	−0.87	3.77
case 12—center	94.82	90.15	−5.79	2.12
case 12—landmarks	94.85	90.21	−5.79	2.09
case 9—center	93.61	87.99	−1.86	3.55
case 9—landmarks	93.60	87.98	−1.88	3.60

## Data Availability

The dataset with paired TRUS and MRI images used for this study is publicly available on ZENODO [21]. The data for the PROMISE12 [14,15] and the SAML [22,23] challenges are also publicly available.

## Abbreviations

The following abbreviations are used in this manuscript:

ASSD	average symmetric surface distance
CT	computed tomography
DAF	deep attentional features
DL	deep learning
*DSC*	dice similarity coefficient
HD	Hausdorff distance
MRI	magnetic resonance imaging
*RVD*	relative volume difference
SSD	sum of squares of intensity differences
TRUS	transrectal ultrasound
UA	prostate apex
UB	prostate base
US	ultrasound
VM	prostate verumontanum
*VOE*	volume overlap error

## Appendix A. Geometric Transformations

Translation ($l_{x}$, $l_{y}$):

(A1) $$\left\{\begin{array}{c} x^{\prime }=x+l_{x}\\ y^{\prime }=y+l_{y} \end{array}\right.$$

Rotation (*r*):

(A2) $$\left\{\begin{array}{c} x^{\prime }=x\cdot cos\left(r\right)-y\cdot sin\left(r\right)\\ y^{\prime }=x\cdot sin\left(r\right)+y\cdot cos\left(r\right) \end{array}\right.$$

Linear tapering along *y*-axis (*t*):

(A3) $$\left\{\begin{array}{c} x^{\prime }=x\cdot \left(\frac{t\cdot y}{a_{y}}+1\right)\\ y^{\prime }=y \end{array}\right.$$

Circular bending along the *y*-axis (*b*):

(A4) $$\left\{\begin{array}{c} x^{\prime }=\left(\frac{a_{y}}{b}-y\right)\cdot sin\left(\frac{x}{\frac{a_{y}}{b}-y}\right)\\ y^{\prime }=\frac{a_{y}}{b}-\left(\frac{a_{y}}{b}-y\right)\cdot cos\left(\frac{x}{\frac{a_{y}}{b}-y}\right) \end{array}\right.$$

## Appendix B. Inverse Transformations

Inverse translation ($l_{x}$, $l_{y}$):

(A5) $$\left\{\begin{array}{c} x=x^{\prime }-l_{x}\\ y=y^{\prime }-l_{y} \end{array}\right.$$

Inverse rotation (*r*):

(A6) $$\left\{\begin{array}{c} x=+x^{\prime }\cdot cos\left(r\right)+y^{\prime }\cdot sin\left(r\right)\\ y=-x^{\prime }\cdot sin\left(r\right)+y^{\prime }\cdot cos\left(r\right) \end{array}\right.$$

Inverse linear tapering along *y*-axis (*t*):

(A7) $$\left\{\begin{array}{c} x=\frac{x^{\prime }}{\frac{t\cdot y}{a_{y}}+1}\\ y=y^{\prime } \end{array}\right.$$

Inverse circular bending along the *y*-axis (*b*):

(A8) $$\left\{\begin{array}{c} x=-sign\left(b\right)\cdot arctan\left(\frac{x^{\prime }}{y^{\prime }-c}\right)\cdot \sqrt{{\left(x^{\prime }\right)}^{2}+{\left(y^{\prime }-\frac{a_{y}}{b}\right)}^{2}}\\ y=\frac{a_{y}}{b}-sign\left(b\right)\cdot \sqrt{{\left(x^{\prime }\right)}^{2}+{\left(y^{\prime }-\frac{a_{y}}{b}\right)}^{2}} \end{array}\right.$$
